# Assessment of Motor Symptoms and Functional Impact in Prodromal and Early Huntington Disease

**DOI:** 10.1371/currents.RRN1244

**Published:** 2011-07-11

**Authors:** Anthony L Vaccarino, Terrence Sills, Karen E. Anderson, Kevin Biglan, Beth Borowsky, Joseph Giuliano, Mark Guttman, Aileen K Ho, Christopher Kennard, Peter Kupchak, G. Bernhard Landwehrmeyer, Andrew Michell, Jane S. Paulsen, Ralf Reilmann, Daniel P van Kammen, John H. Warner, Ken Evans

**Affiliations:** ^*^Research Methods, Ontario Cancer Biomarker Network, Toronto, Ontario, Canada; ^‡^Department of Psychiatry and Department of Neurology, University of Maryland, School of Medicine, Baltimore, MD USA; ^§^Department of Neurology, University of Rochester, Rochester, NY, USA; ^¶^Translational Medicine, CHDI Foundation, Inc., Princeton NJ; ^#^CHDI Foundation, Inc.; ^**^Division of Neurology, Department of Medicine, University of Toronto, Toronto, Ontario Canada; ^††^School of Psychology and Clinical Language Sciences, University of Reading, U.K.; ^‡‡^Nuffield Department of Clinical Neurosciences, University of Oxford, Oxford, United Kingdom; ^¶¶^University of Ulm, Ulm, Germany; ^##^Dept of Clinical Neuroscience, Cambridge University Hospitals, UK; ^***^Department of Psychiatry, The University of Iowa Carver College of Medicine, Iowa City, IA, USA; ^†††^University of Muenster, Dept. of Neurology; ^‡‡‡^CNS Drug Development consultant and ^§§§^Department of Biostatistics, CHDI Foundation, Princeton NJ USA

## Abstract

The Functional Rating Scale Taskforce for pre-Huntington Disease (FuRST-pHD) is a multinational, multidisciplinary initiative with the goal of developing a data-driven, comprehensive, psychometrically sound, rating scale for assessing symptoms and functional ability in prodromal and early Huntington disease (HD) gene expansion carriers. The process involves input from numerous sources to identify relevant symptom domains, including HD individuals, caregivers, and experts from a variety of fields, as well as knowledge gained from the analysis of data from ongoing large-scale studies in HD using existing clinical scales. This is an iterative process in which an ongoing series of field tests in prodromal (prHD) and early HD individuals provides the team with data on which to make decisions regarding which questions should undergo further development or testing and which should be excluded. We report here the development and assessment of the first iteration of interview questions aimed to assess functional impact of motor manifestations in prHD and early HD individuals.

## Introduction

Earliest clinical manifestations of Huntington disease (HD) are poorly characterized, and there is a need for clinical scales specifically designed to measure early changes in HD gene expansion carriers. The Functional Rating Scale Taskforce for pre-Huntington Disease (FuRST-pHD) is a multinational, multidisciplinary collaboration to develop a valid functional rating scale to assess changes in symptom severity in HD gene expansion carriers who do not yet meet criteria for a formal clinical diagnosis (prodromal HD or prHD) or are early manifest.[1] Such a measurement tool is essential to better understand the earliest manifestations of HD and to evaluate novel therapies early in the course of disease. 

FuRST-pHD has established an inclusive process using input from numerous sources, including prHD and early HD individuals, caregivers, and experts from a variety of fields, as well as from ongoing large-scale HD studies using existing clinical scales.[1] As part of the process, an inclusive series of “Working Groups” of individuals with clinical and/or scale development expertise have been established to review existing data and develop interview questions within the specific domain under study. Once these interview questions are developed, they are distributed to trained raters for beta testing in prHD and early HD individuals. This is an iterative process, in which changes or deletions (as appropriate) are made based on empirical evidence obtained during field testing; the modified questions are then tested during subsequent iterations so that the list can ultimately be winnowed to select optimal items for scale inclusion. We report here the development and assessment of the first iteration of interview questions aimed to assess functional impact of motor manifestations in prHD and early HD individuals.

## Methods 

A two-day Motor Symptoms Working Group meeting was held in Toronto, Canada, January 19–20, 2009. The working group charge was to review available evidence and develop interview questions to assess functional impact of motor signs and symptoms.

### Evidence Reviewed

#### UHDRS Motor Subscale.

The Unified Huntington's Disease Rating Scale (UHDRS) Motor Subscale was developed for the clinical assessment of motor signs of HD and is the current "gold-standard" outcome measure used in clinical trials of HD. In developing interview questions, the Working Group reviewed retrospective data from two multicentre, longitudinal studies in prHD (PREDICT-HD) and HD (REGISTRY).[2] As expected there were differences in the severity of UHDRS motor signs in prHD and HD. In prHD, overall severity of UHDRS motor signs were low, and participants had scores of 2 or above on very few items. However, there were indications that some items scored higher than others, including saccades, ocular pursuit, and finger tapping. In HD, motor items that assessed ocular pursuit, saccade initiation, finger tapping, tandem walking, and to a lesser extent saccade velocity, dysarthia, tongue protrusion, pronation/supination, Luria, bradykinesia, chorea, gait, and balance on the retropulsion test were found to discriminate individual differences across a broad range of motor severity.[2]

Although these data clearly indicate that some motor signs assessed by the UHDRS are present even at early stages of HD, the degree that these motor signs are clinically meaningful has not been determined. Current data would benefit from evidence indicating that UHDRS assessments of motor signs are associated with symptoms that can be perceived by the patient (and therefore also the clinician) and interfere with their day-to-day functioning. To this end, it was agreed that some of the interview questions would be developed to assess the potential functional impact of these motor signs. 

#### Patient and Companion Input.

The FDA views input from participants, caregivers, and family members as an essential element in developing valid clinical assessment tools.[3] To ensure that the scale reflects concepts that are important from the participant's perspective, patient/companion focus groups were held to identify early symptoms experienced by HD gene carriers and to determine the functional impact of these symptoms. The focus groups were held in a number of countries using the local languages (France, Netherlands, United Kingdom, United States, Portugal, and Spain) with all participants (prHD, early HD and companions) being asked a series of open-ended questions related to symptom occurrence in prHD. All focus group sites had IRB/EC approval, and all participants provided informed consent. These data were used to maximise symptom assessment and were considered in development of the interview questions. The following motor-related symptoms/problems were reported by the focus groups:

*Head twitch; Losing balance; Hand writing; Gait; Fine motor; Choking; Hand-eye coordination; Twitching; Fidgety; Dropping things; Restless feet; Chorea; Tics; Clumsiness; Falling; Movements during sleep; Tremors; Eye tracking; Walking difficulty as a couple; Rigidity of muscle; Tension in face; Instability; Uncontrolled movements.*

#### Expert Opinion and Experience of Participants.

In addition to reviewing existing data, working group participant experiences and opinion were also discussed, both within HD and in other movement-related disorders, including Parkinson's disease.

### Development of Interview Questions 

After review of existing data, relevant motor symptom domains were identified, and interview questions were developed to probe the experience of those symptoms (and determine their severity) in the prHD and early HD population. The FuRST-pHD has adopted a semi-structured clinician-administered interview similar to that used for the GRID-HAMD. The GRID format directs the rater to score symptom frequency and intensity separately, while giving them clear scoring anchors, a semi-structured interview guide, and overall definitions. This method has been employed successfully and is user-friendly, with acceptable agreement among independent raters.[4] The working group developed interview questions, including structured interview guides, scoring conventions, scoring anchors, and symptom definitions. Following the meeting, draft interview questions were circulated for comment on a shared internet site (Sharepoint).

Based on a review of the evidence, 11 interview questions were developed for field testing (Table 1).

**Table 1.** Interview Questions Developed by Working Group

**Table:** 

**Interview Question**	**Description/definition**	**Sources of evidence reviewed and considered**
**Reading**	Assesses experiences of problems reading in a general fashion, and not limited to any particular domain (e.g., motor difficulties, cognitive difficulties).	Clinical experience (This item was developed to assess general problems with reading)
**Tracking while reading**	Assesses ability to track (moving from word to word, line to line or maintaining eyes on page) during reading.	UHDRS (Ocular Pursuit, Saccades); Focus groups; Clinical Experience
**Tracking moving objects with eyes**	Assesses ability to follow/track moving objects with eyes, which include ocular pursuits allowing eyes and head to follow moving objects with integrated and coordinated movements.	UHDRS (Ocular Pursuit, Saccades); Focus groups; Clinical Experience
**Balance on one foot**	Assesses ability to perform tasks that require balance on one foot.	UHDRS (Bradykinesia, Dystonia, Chorea, Gait, Tandem Walking, Retopulsion); Focus groups; Clinical Experience
**Balance when walking**	Assesses problems with maintaining balance when walking.	UHDRS (Bradykinesia, Dystonia, Chorea, Gait, Tandem Walking, Retopulsion); Focus groups; Clinical Experience
**Shifting gaze**	Assesses ability to shift gaze from one object to another.	UHDRS (Ocular Pursuit, Saccades); Clinical Experience
**Fine motor ability**	Assesses fine motor ability requiring significant dexterity of the hands and fingers.	UHDRS (Bradykinesia, Dystonia, Chorea, Finger Tapping, Pronate/Supinate, Luria, Rigidity); Focus groups; Clinical Experience
**Complex motor behavior**	Assesses the performance of complex motor behavior.	UHDRS (Bradykinesia, Dystonia, Chorea, Finger Tapping, Pronate/Supinate, Luria, Rigidity); Focus groups; Clinical Experience
**Writing**	Assesses normal adult writing ability, including legibility.	UHDRS (Bradykinesia, Dystonia, Chorea, Finger Tapping, Pronate/Supinate, Luria, Rigidity); Focus groups; Clinical Experience
**Clumsiness**	Assesses clumsiness, including any avoidance of tasks due to clumsiness or changes in behavior to compensate.	UHDRS (Bradykinesia, Dystonia, Chorea, Finger Tapping, Pronate/Supinate, Luria, Ocular Pursuit, Rigidity); Focus groups; Clinical Experience
**Functional impact** ** **	Assess the functional impact of motor deficits (if any reported) on daily activities. Question gathers additional patient-reported descriptive data.	Clinical Experience

### Field Testing of Interview Questions

Field testing of interview questions in prHD (UHDRS Diagnostic Confidence Level < 4) and early HD (within 5 years from onset of clinical motor signs) was conducted within the PREDICT-HD program and at independently contracted sites. All data collection sites had IRB/EC approval, and all participants provided informed consent. Prior to conducting the clinical interview, all raters were trained (via webinar or in person) to ensure that all trainees had an adequate conceptual understanding for administering and scoring each of the items. A minimum sample size of 100 was targeted.

### Data Analysis 

The distribution of the composite score for each individual item was compiled, and summary statistics associated with each item score were computed. Distributions of item scores for HD and prHD subgroups were statistically compared using the non-parametric Mann-Whitney U test.  

Non-parametric item response analyses were performed to determine the relationship between scores on the individual interview questions and total score. Item Response Theory has been demonstrated to be useful in evaluating the performance of individual items (symptoms) on rating scales, by assessing the relationship between a score assigned to an item and the overall severity of the disease.[2] [5] IRT software (TESTGRAF) was used to generate Option Characteristic Curves (OCCs) that display the probability of a particular option score (i.e., a score of 0, 1, 2, 3, 4) on each Interview Question as a function of overall level of severity. In the present analyses, total motor item score was used as a measure of severity. To illustrate this, Figure 1 depicts a hypothetically ‘‘ideal’’ item from an item response perspective, which is characterized by a clear identification of the range of severity scores over which an option is most likely to be endorsed, rapid changes in the curves that correspond to changes in severity, and an orderly relationship between the weight assigned to the option and the region of severity over which an item is likely to be endorsed. As such, OCCs provide a graphical representation of how informative a particular item (or symptom) is as a measure of illness. Frequency distribution of option scoring within each interview question were also generated.

**Figure:**
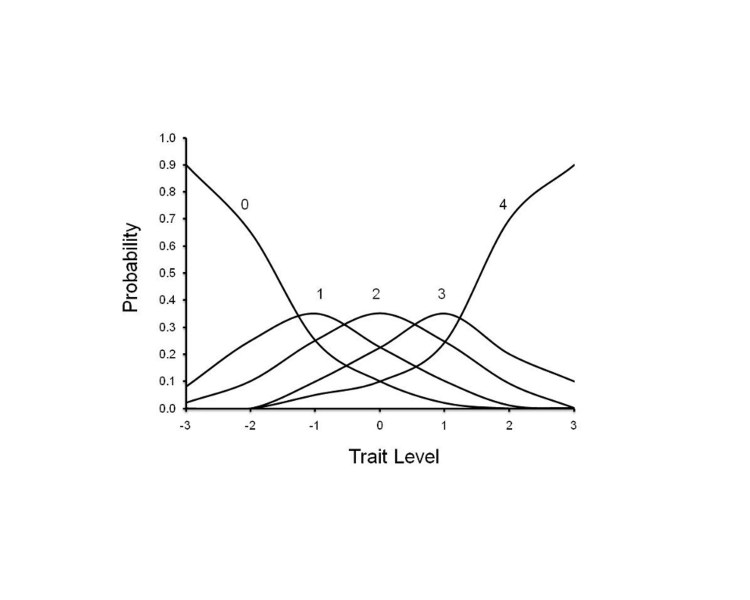
**Figure 1**. OCC for a hypothetically ‘‘ideal’’ item. The OCC shows the probability (*y*-axis) of endorsing a particular option for the item at different levels of the trait (*x*-axis).

Interview questions which were found to produce scoring and discrimination across ranges of overall severity were putatively selected for further testing. Scores for prHD and HD subjects were computed and compared statistically using the Mann-Whitney U test. The measure of internal consistency of the subscale was estimated using Cronbach's alpha, and item-total correlations between subscale scores and scores of individual questions not included in the subscale were computed.  

## Results

A total of 131 CRFs were completed. The participant demographic characteristics are shown in Table 2.

**Table 2.** Demographic Characteristics

**Table:** 

** **	**All Subjects**	** prHD**	** HD**
**Sample size**	N=131	N=73 (56%)	N=58 (44%)
**Male gender**	N=56 (43%)	N=26 (36%)	N=30 (52%)
**Age**	48.0 (18-73)	45.0 (18-71)	51.9 (23-73)

A follow-up Motor Working Group meeting was held (via webinar) to review data and make recommendations in moving forward, including item deletion and modification/refinement. The FDA PRO Guidance was used to guide the decision making process.[3]

OCCs and scoring frequency distributions were generated for each of the interview questions. Of the 11 tested, 7 interview questions were found to produce scoring and discrimination across ranges of overall severity:

### ✓  Balance on one foot

### ✓  Balance when walking

### ✓  Fine motor ability

### ✓  Complex motor behavior

### ✓  Writing

### ✓  Clumsiness

### ✓  Functional impact

**

The internal consistency of these seven items was very high, as were the corrected item-total correlations (Table 3, shaded rows). Cronbach's alpha was 0.93 with respect to the entire study population, 0.89 with respect to the prHD subgroup, and 0.93 with respect to the HD subgroup. All corrected item-total correlations were 0.69 or higher with respect to the HD subgroup; the only items displaying a low item-total correlation with respect to the prHD subgroup were the "Fine motor ability" item and the remainder (Table 3). 

****

**Figure:**
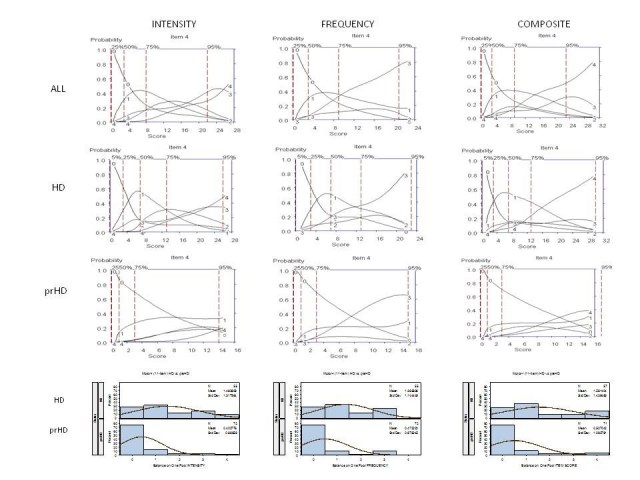
**Figure 2.** OCCs and Frequency distribution for interview question assessing ability to perform tasks that require balance on one foot for all participants, HD and prHD. Data is presented separately for symptom intensity (left row), frequency (middle row), and composite (right row) scores.

The mean total composite score with respect to the above 7 items was 2.55 in prHD subjects and 8.98 in HD subjects; the difference in mean scores was statistically significant (*p* < 0.001, Mann-Whitney U test). In addition, significant differences were noted in scoring between prHD and HD for each of the individual items, with greater intensity and frequency of symptoms noted in early HD participants (*p* < 0.001 with respect to each item, Mann-Whitney U test) (Table 4). This is not surprising given that the classification of prHD is defined as the absence of sufficient motor signs to meet conventional diagnostic criteria for manifest disease.[6] For example, in assessing ability to perform tasks that require balance on one foot, scoring was driven primarily by early HD participants (Figure 2, Table 4). In prHD, the option with the highest probably of being scored for symptom intensity increased from "0" to "1" (mild: sense of unsteadiness/imbalance on one foot that others may not notice, but no impact on function of daily activities) suggesting that although these changes are noticeable to the participant, the functional impact may be minimal. By contrast, in early HD participants scores of "3" (severe: needs support to maintain balance) and "4" (very severe: cannot stand on one foot, must sit down to do things) were sometimes endorsed. The poor discrimination between symptom intensity scores of "3" and "4" indicates that modifications to the wording of these options would be needed to allow for better discrimination. 

****

**Table 3.  **Correlations between Interview Questions Scores

**Table:** 

Item	Item-total correlation (all subjects)	Item-total correlation (prHD subjects)	Item-total correlation (HD subjects)
Reading	0.389	0.340	0.360
Tracking while reading	0.489	0.202	0.639
Tracking objects with eyes	0.169	0.373	0.190
Balance on one foot	0.758	0.785	0.690
Balance when walking	0.858	0.817	0.857
Shifting gaze	0.289	0.333	0.349
Fine motor ability	0.718	0.257	0.774
Complex motor behavior	0.722	0.532	0.733
Writing	0.820	0.805	0.776
Clumsiness	0.795	0.744	0.774
Functional impact	0.858	0.902	0.828

**
 Table 4.** Summary of Mean Composite Scores

**Table:** 

**Item**	**Overall mean score **	**prHD mean score**	**HD mean score**
Reading	0.33	0.21	0.48
Tracking while reading	0.29	0.22	0.37
Tracking objects with eyes	0.08	0.11	0.03
Balance on one foot	0.98	0.51	1.56
Balance when walking	0.76	0.44	1.17
Shifting gaze	0.12	0.12	0.12
Fine motor ability	0.66	0.16	1.28
Complex motor behavior	0.50	0.18	0.90
Writing	0.96	0.49	1.55
Clumsiness	0.88	0.55	1.29
Functional impact	0.80	0.44	1.24

It was agreed that these 7 interview questions would be modified accordingly for testing in subsequent iterations; examination of the OCCs provided data on which modifications should be made to improve item performance, including changes in wording and scoring options.

The remaining 4 questions were very rarely endorsed (see Table 4, non-shaded items), and a score of zero had the highest probability of being scored across most of the range of severity (Figure 3, as example): 

### ✗  Reading

### ✗  Tracking while reading

### ✗  Tracking moving objects with eyes

### ✗  Shifting gaze

**

The low frequency of response (Table 4) and generally poor discriminative properties (Figure 3) limit the usefulness of these interview questions for assessing motor manifestations in prHD and early HD. In general, the correlations between each of these 4 individual item composite scores and the total score from the 7 highly-endorsed items were low; "Tracking while reading" correlated highly with the motor subscale score in the subset of HD subjects (Table 3), but was nonetheless rarely endorsed (Table 4). 

**

**Figure:**
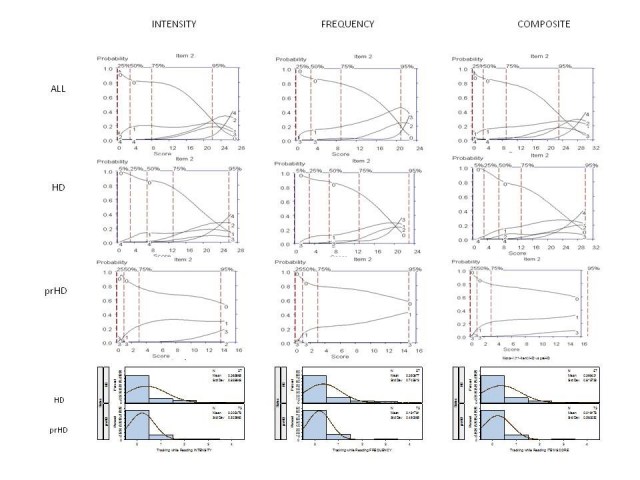
**Figure 3. **OCCs and frequency distribution of a representative rarely endorsed interview question (assessing ability to track while reading) for all participants, HD and prHD. Data is presented separately for symptom intensity (left row), frequency (middle row), and composite (right row) scores.

****It was agreed that these 4 interview questions should be removed from subsequent iterations on the basis of Relevance (Reported as not relevant by a large segment of the population of interest) and Response Range (A high percentage of patients respond at the floor) as outlined in Table 1 of the FDA PRO Guidance.[3]

## Discussion

As part of FuRST-pHD, interview questions are being developed to assess symptoms and functional ability in prodromal and early HD gene expansion carriers. We report here the development and beta testing of first iteration interview questions designed to assess motor-related changes and functional impact. 

These data suggest that although UHDRS motor signs may indeed be present (see Table 1), the functional impact of some of these motor signs may be minimal in prHD and early HD. For example, ocular pursuit dysfunction is associated with difficulty in visually tracking or following objects, as well as problems with reading, including loss of place and repetition (COVD, 2009). In the present study, however, very few participants reported related problems (i.e., tracking while reading, tracking moving objects, and shifting gaze, see Figure 3, Table 4) despite the presence of significant deficits in ocular pursuit and saccades in prodromal and early stages of HD.[2] [7] [8]

On the other hand, although the presence of some UHDRS motor signs may have minimal impact in the populations tested (i.e., ocular pursuit and saccades), other motor signs may indeed be associated with symptoms that can be perceived and may interfere with day-to-day functioning. For example, problems in the ability to perform tasks that require balance on one foot (see Figure 2, Table 4) may reflect the presence of motor signs assessed in the UHDRS such as Bradykinesia, Dystonia, Chorea, Gait, Tandem Walking, and/or Retopulsion. Indeed, as the severity of these UHDRS motor signs increase from prHD to HD so did the severity of symptoms that assessed the potential functional impact of these signs. It is also important to note that many symptoms reported as problems in prHD by focus groups, including losing balance, hand writing, gait, fine motor, hand-eye coordination, dropping things, clumsiness, falling, walking difficulty, and instability were also assessed and reported as problematic in the present study (see Table 1). 

Based on the results, seven interview questions have been selected for further testing, have been modified accordingly by the working group, and are currently undergoing a second iteration of field testing. The results of the second iteration will be reported once completed. 

### Acknowledgments 

CHDI Foundation, Inc. – a not-for-profit research organisation whose mission is to rapidly and collaboratively discover and develop therapies that slow the progression of Huntington’s disease – initiated and sponsored the development of the FuRST-pHD. We thank Jamie Levey for her help coordinating the European focus groups, LaVonne Goodman for her help coordinating the USA focus groups, and Stacie M Vik and Barbara McQuaid for administrative assistance.

### Funding Information

FuRST-pHD is funded by CHDI. PREDICT-HD is supported by the National Institutes for Health, National Institute of Neurological Disorders and Stroke (NS40068) and CHDI Foundation, Inc

### Competing Interests

The authors have declared that no competing interests exist.

### FuRST-pHD Core Team

K Anderson, B Borowsky, K Evans, J Giuliano, M. Guttman. A Ho, JS Paulsen, T Sills, A Vaccarino, D van Kammen

### Motor Symptoms Working Group

FuRST-pHD Core Team, K Biglan, C Kennard, B Landwehrmeyer, A Michell, R Reilmann, J Warner

### Statistics

S Gilbert-Evans, P Kupchak, T Sills, A Vaccarino 

### Contributing Field Testing Sites and Coordinators

PREDICT-HD: Baylor College of Medicine, Houston, Texas, USA (Joseph Jankovic, MD, Christine Hunter, RN, CCRC, and William Ondo, MD); OCBN Contracted: Birmingham and Solihull Mental Health, Birmingham, UK (Hugh Rickards, MD, Jenny Crooks, BA, Jan Wright, BA); Center for Movement Disorders, Markham, Ontario, Canada (Mark Guttman, MD, Irita Karmalkar, BA, Alanna Sheinberg, BA, and Adam Singer, BA); University of Melbourne, AU (David Ames, MD, Edmond Chiu, MD, Phyllis Chua, MD, Olga Yastrubetskaya, PhD, Joy Preston, Anita Goh, D.Psych, and Angela Komiti, BS, MA); University of Iowa, Iowa City, IA, USA (Leigh Beglinger, PhD, Thomas Wassink, MD, Patricia Ryan, MSW, MA, Stephen Cross, BA, Mycah Kimble, BA, Stacie Vik, BA); Huntington Disease Drug Works, Seattle, WA, USA (LaVonne Goodman, MD); North York General Hospital, Toronto. Ontario, Canada (Clare Gibbons, MS, Jeanne Kennedy, BScNEd, RN, and Wendy Meschino, MD)

### Focus Groups

EHDN Language Area Coordinators Portugal (J Ferreira, T Mestre), Spain (A Martínez Descals), France (A Durr, C Jauffret), The Netherlands (R Bos, R Roos, M-N Witjes-Ané), UK (R Fullam, O Handley, J Naji); HD Drug Works, Seattle, USA (L Goodman) 

### Corresponding Author

Anthony L Vaccarino, avaccarino@ocbn.ca
